# Fluorination of vertically aligned carbon nanotubes: from CF_4_ plasma chemistry to surface functionalization

**DOI:** 10.3762/bjnano.8.173

**Published:** 2017-08-21

**Authors:** Claudia Struzzi, Mattia Scardamaglia, Jean-François Colomer, Alberto Verdini, Luca Floreano, Rony Snyders, Carla Bittencourt

**Affiliations:** 1 Chimie des Interactions Plasma-Surface, CIRMAP, University of Mons, 7000 Mons, Belgium; 2 Research Group on Carbon Nanostructures (CARBONNAGe), University of Namur, 5000 Namur, Belgium; 3 CNR-IOM, Laboratorio Nazionale TASC, I-34149 Trieste, Italy; 4 Materia Nova Research Center, 7000 Mons, Belgium

**Keywords:** carbon nanotubes, CF_4_ plasma, fluorination, plasma chemistry, surface chemistry

## Abstract

The surface chemistry of plasma fluorinated vertically aligned carbon nanotubes (vCNT) is correlated to the CF_4_ plasma chemical composition. The results obtained via FTIR and mass spectrometry are combined with the XPS and Raman analysis of the sample surface showing the dependence on different plasma parameters (power, time and distance from the plasma region) on the resulting fluorination. Photoemission and absorption spectroscopies are used to investigate the evolution of the electronic properties as a function of the fluorine content at the vCNT surface. The samples suffer a limited ageing effect, with a small loss of fluorine functionalities after two weeks in ambient conditions.

## Introduction

Tetrafluoromethane (CF_4_) plasma emerged as a strategic tool when exploiting the ability of CF_x_ radicals to promote etching of a variety of substrates frequently used in the manufacturing of microelectronic devices [1]. In the CF_4_ plasma, CF_x_ radicals are primarily produced by electron-impact induced dissociation. In this framework, attention has been dedicated to the analysis of fluorine-based plasma chemical composition, and many diagnostic techniques have been used to understand the possible reactions occurring during pure CF_4_ discharge [2–4]. The study of the CF_4_ plasma chemistry has been performed focusing on the evaluation of the lifetime of plasma species and density distribution [5–6], considering the reaction with the walls of the chamber [7], and determining the role of molecular oxygen [8–10] or hydrogen [11] in the gas mixture. Progressively, the emerging demand in functionalizing the surface of carbon-based materials has led to the use of various atoms for the tuning of the surface properties [12–16] and fluorine-based gas precursors have also been employed, including CF_4_ [17], with special attention to their effect on carbon nanotubes [18–21]. This carbon allotrope exhibits a low-reactive surface; therefore, plasma parameters can be adjusted to promote a controlled fluorination, avoiding the chemical etching of carbon atoms from the sample. The fluorination entails the conversion of the sp^2^ hybridization into sp^3^ and makes the surface highly hydrophobic: the introduction of polar groups has been successfully adopted to initiate subsequent functionalization [22] and to profitably implement the fluorinated carbon nanomaterials in numerous applications, including gas sensors for hindering the moisture interference [23–24]. In single- and multiwalled nanotubes the tuning of surface properties via fluorine-based plasma has been investigated highlighting the modification of morphology, chemical composition and electronic properties, as well as focusing on the field emission performances and the thermal stability [25–27]. However, a combined study including the effect of the plasma treatment at the carbon nanotubes surface and the fundamental processes involving the production of CF_x_ radicals and ions is lacking. Additionally, molecules as water or residual oxygen in the background pressure of the functionalization chamber may be activated by the plasma and recombination with oxygen or fluorine species can occur, leading to COF_x_ formation affecting the functionalization of the carbon nanotubes.

In this work, we correlate the chemical composition of the CF_4_ plasma with changes occurring on vertically aligned carbon nanotubes (vCNT) exposed to the fluorine-rich glow discharge. In situ residual gas analysis (RGA) mass spectrometry is performed to probe the chemistry of the plasma phase and to individuate the plasma products that are simultaneously explored by Fourier transform infrared spectroscopy (FTIR). The combination of these experimental approaches enables evaluating the nature of the species that are generated inside the glow considering the presence of contaminants in the background pressure and at the walls of the functionalization chamber. These contaminants are responsible for the CO, CO_2_ and COF_2_ production due to reactions occurring within the plasma region. FTIR analysis reveals the absence of COF fragments inside the discharge, as these fragments rapidly react with oxygen to produce CO_2_ or they are dissociated to yield CO, and therefore the fluorination of the vCNT is not affected. Furthermore, XPS analysis shows an oxygen concentration in the plasma functionalized vCNT similar to the one detected on the pristine vCNT surface, thus indicating that the oxygen-containing species are not grafted on the vCNT. Therefore, surface contamination can be limited or avoided if the sample is not immersed in the discharge.

XPS and Raman spectroscopies are used to evaluate also the correlation between the plasma applied power and the amount of defective sites created at the vCNT surface: a higher fragmentation rate of the parent CF_4_ molecule is achieved by increasing the plasma power, consequently smaller and lighter radicals and ions are produced. These ions cause less damage compared to the larger ones produced in low power plasmas. Comparing the effect of the treatment duration, an increased production of defective sites is observed for short plasma treatment: under this condition a lower functionalization yield is obtained and the non-saturated defective sites are quenched by oxygen after air exposure. On the contrary, defects are saturated by fluorine atoms during longer plasma treatment, in agreement with the corresponding increase in the F content detected at the surface. The evolution of the electronic properties of fluorinated vCNT is discussed as a function of the fluorine content and ageing effects are verified after storing the samples for two weeks under ambient conditions.

## Results and Discussion

**Figure 1:**
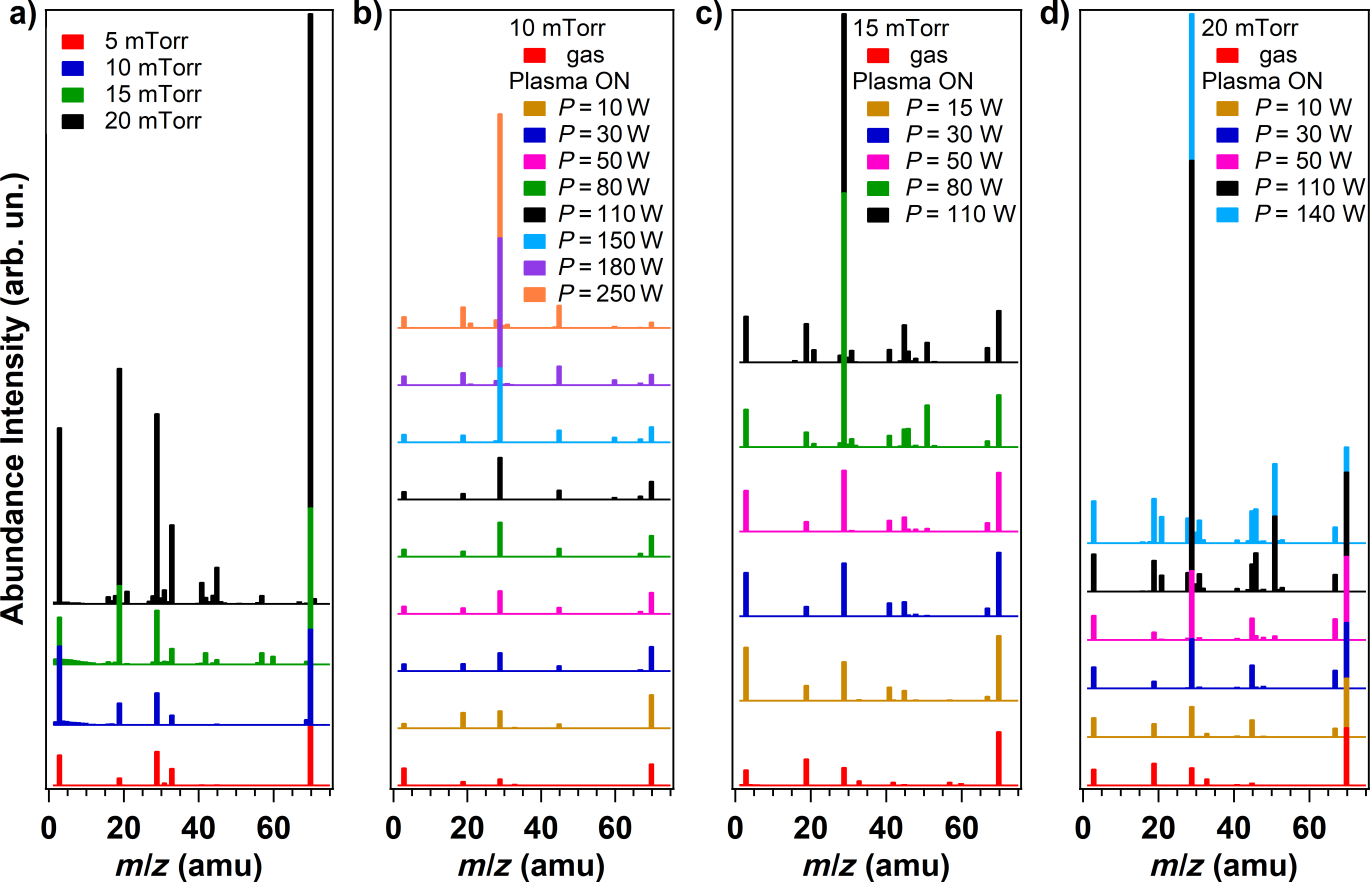
Mass spectra recorded at a) different gas pressures (5, 10, 15 and 20 mTorr) and at different power values using a fixed working pressure of: b) 10 mTorr, c) 15 mTorr and d) 20 mTorr, respectively.

In the present work, we combine the study of the CF_4_ plasma chemistry with the analysis of the plasma treatment effects on the surface chemistry of the vCNT. The mass and the vibrational spectra of the precursor gas are collected as a function of different plasma parameters. The residual gas analysis (RGA) mass spectroscopy data are illustrated in Figure 1, where the evolution of the ions signals is reported for: a) different pressures of CF_4_ gas flow (5, 10, 15 and 20 mTorr); different power values, ranging from 10 to 250 W, at a fixed working pressure of 10 mTorr (b), 15 mTorr (c) and 20 mTorr (d). The principal fragments detected are associated to the following ionized molecules: H_2_O (*m*/*z* = 18 amu), HF (*m*/*z* = 20 amu), CO (*m*/*z* = 28 amu), CF (*m*/*z* = 31 amu), O_2_ (*m*/*z* = 32 amu), CO_2_ (*m*/*z* = 44 amu), COF (*m*/*z* = 47 amu), CF_2_ (*m*/*z* = 50 amu), COF_2_ (*m*/*z* = 66 amu) and CF_3_ (*m*/*z* = 69 amu). Since only charged species can be detected by mass spectrometry, neutral species have to be ionized in the source of the spectrometer by means of fast moving electrons and the electron ionization can contribute to partial fragmentation, as observed in Figure 1a. When the CF_4_ gas flows in the chamber and the plasma power is set to zero, the origin of the ionized molecules is related to the dissociative ionization occurring in the mass spectrometer apparatus. However, as soon as the plasma discharge is initiated, the discrimination between the ionized species arising from the plasma and the species arising from dissociative ionization in the mass spectrometer is not straightforward.

Among the working pressure values investigated, we select 10 mTorr for the detailed analysis of the plasma chemical composition and the concomitant functionalization of the vCNT. The working pressure of 10 mTorr represents the best experimental condition because the plasma discharge shows stability for a larger range of applied power values, thus allowing us to focus on the fragmentation of CF_4_ within a more extended range varying from 10 to 250 W.

**Figure 2:**
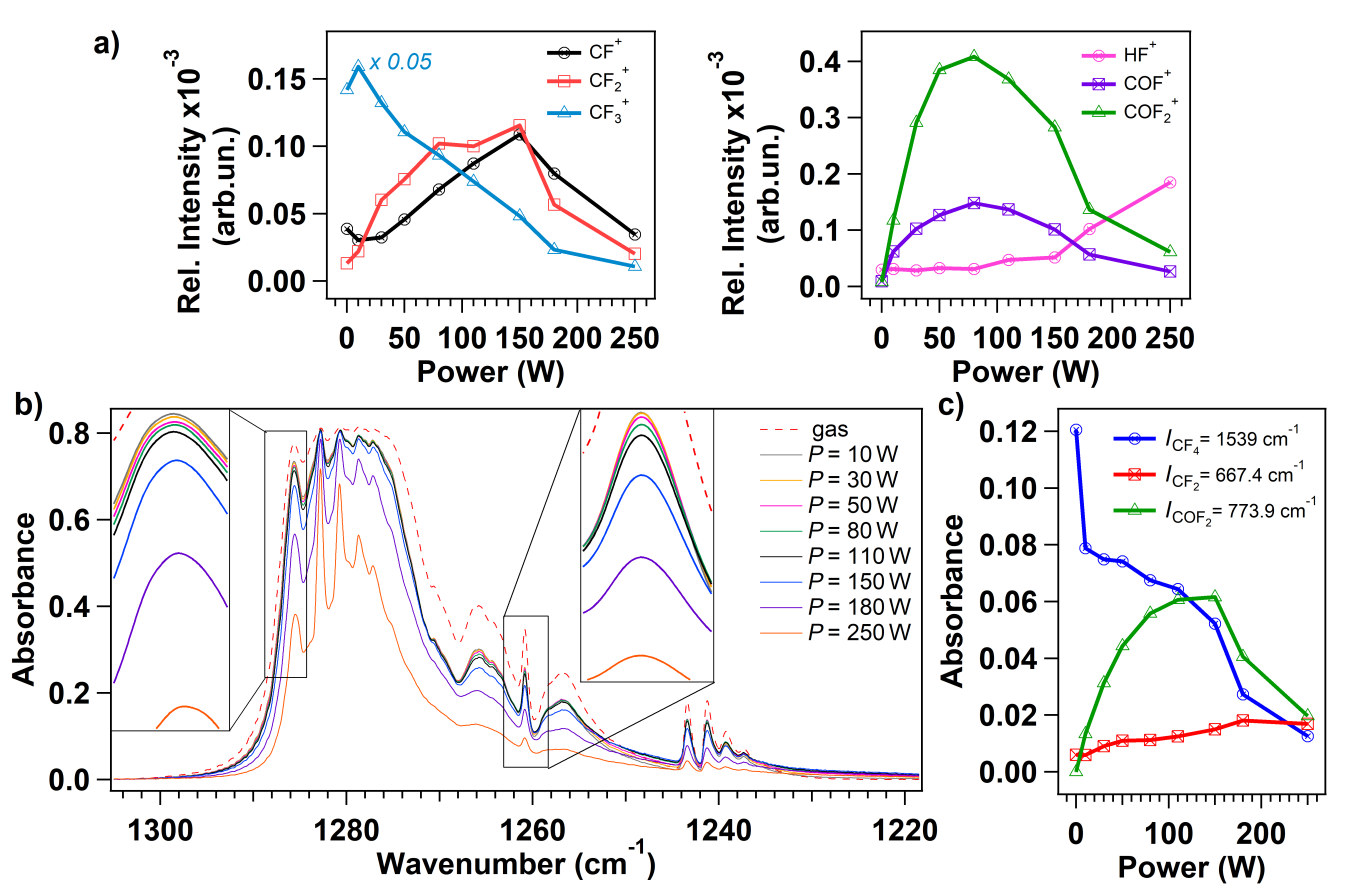
a) Evolution of the normalized ion signals, extracted from the mass spectra at different plasma power (the working pressure is set to 10 mTorr). The masses under study are relative to the following ionized species: CF (*m*/*z* = 31 amu), CF_2_ (*m*/*z* = 50 amu), CF_3_ (*m*/*z* = 69 amu), and HF (*m*/*z* = 20 amu), COF (*m*/*z* = 47 amu), COF_2_ (*m*/*z* = 66 amu). b) FTIR spectra acquired during pure CF_4_ gas phase (dotted line) and during CF_4_ plasma ignited at different power (from 10 to 250 W). The two frames in the figure detail the peaks located at 1286 and 1261 cm^−1^ for better visualization of the intensity variation. c) Intensities of the FTIR peaks related to CF_4_ (at 1539 cm^−1^), CF_2_ (at 667.4 cm^−1^) and COF_2_ (at 773.9 cm^−1^) are shown for different power used to initiate the discharge.

In Figure 2a we show the relative intensity of the measured ion currents corresponding to the products of interest [4]. The relative intensity is defined as the intensity normalized to the total ion signal of all masses detected during each experiment. The point relative to power *P* = 0 W is representative for the CF_4_ gas phase, the plasma is not ignited and the CF_4_ flows in the chamber. The mass spectrum is dominated by the intense signal corresponding to the ionized CF_3_ (*m*/*z* =69 amu) and to the lower signals relative to CF_2_ (*m*/*z* = 50 amu) and CF (*m*/*z* = 31 amu) ionized fragments, suggesting that dissociative ionization of the parent CF_4_ molecule occurs in the ionization chamber of the RGA apparatus. The CF_4_ is not detected due to its low stability, as already reported in [4]. As soon as the plasma is initiated, new signals are detected in correspondence to *m*/*z* = 20, 47 and 66 amu, indicating the production of ionized HF, COF and COF_2_, respectively. Simultaneously, three-bodies reactions and recombination at the walls occur as noticeable by the production of CO and CO_2_, in agreement with previous works [8–9], in detriment of water and oxygen that are present in the background pressure of the chamber (see Figure S1 in Supporting Information File 1). For increasing applied power, the CF_3_ species are further fragmented in CF_2_ and CF species that may react with plasma-activated background contaminants in the vacuum chamber, thus generating COF and COF_2_ as well. The signal corresponding to ionized HF raises for applied power higher than 110 W, in correspondence to the intensities depletion of ionized CF_2_, CF, COF and COF_2_, suggesting a high fragmentation rate that can be associated to the higher energy supplied to charged particles that boost the fragmentation increasing the density of fluorine radicals.

Complementary to mass spectrometry, FTIR spectroscopy analysis is performed under different plasma power conditions to evaluate if COF_x_ species are present in the CF_4_ plasma discharge or they are formed due to ionization inside the mass spectrometer; the full range FTIR spectra are shown in Figure S2 in Supporting Information File 1. The characteristic region of the C–F bond stretching vibration in FTIR spectra ranges from 1218 to 1310 cm^−1^ (Figure 2b) and it is dominated by a main triply degenerate stretching mode υ_3_ of CF_4_ located at 1283 cm^−1^ [2–3]. The intensity of the peaks related to CF_4_ slightly decreases when increasing the power during the plasma discharge ignition (Figure 2b). For an applied power higher than 110 W, the intensity of the modes in the 1278–1287 cm^−1^ region reduces further loosing nearly 50% of the initial absorbance intensity when an applied power of 250 W is used. The two frames in Figure 2b detail the peaks located at 1261 and at 1286 cm^−1^. The peak at 1261 cm^−1^ is associated to the vibration mode of CF_3_ radical [11,28] and its evolution with power tracks the intensity decrease of the corresponding ion signal shown in Figure 2a.

For a straightforward analysis, the absorbance intensities of three isolated modes are studied as a function of the plasma power (Figure 2c): the modes at 677.4 cm^−1^ and 773.9 cm^−1^ are associated respectively to the bend mode of CF_2_ and to the out-of-plane deformation mode of COF_2_, while the peak at 1539 cm^−1^ is related to CF_4_ as a combination of υ_1_ (at 904 cm^−1^) and υ_4_ (at 631 cm^−1^) modes [29]. The decrease in the absorbance of CF_4_ vibrational mode corresponds to the increase in the CF_2_ and COF_2_ vibrational modes intensities testifying the fragmentation of the parent molecule. The absorbance intensity of the COF_2_ vibrational mode precisely follows the ion signal corresponding to the ionized COF_2_ (Figure 2a): it increases until the plasma power reaches approximately 110 W, and then it decreases due to conversion process taking place with other species in the discharge. On the other hand, the absorbance intensity trend of the CF_2_ vibration doesn’t show correspondence with its relative signal acquired with the mass spectrum: reactions involving this fragment may occur at higher powers just outside the core of the discharge, where the vibrational modes can not be measured with the IR beam. This mechanism is confirmed by the detection of a low intensity signal in the mass spectrum in correspondence to CF and CF_2_: being highly reactive species, they are likely to react with other radicals in the discharge before reaching the mass spectrometer apparatus which is located around 30 cm away from the coil. No vibrational modes are observed in correspondence of COF (expected at 633 cm^−1^), this result confirms that the COF fragment is produced by dissociative ionization in the mass spectrum as, inside the discharge, it is rapidly reacting with oxygen to produce CO_2_ or it is dissociated to yield CO, as suggested previously [9].

**Figure 3:**
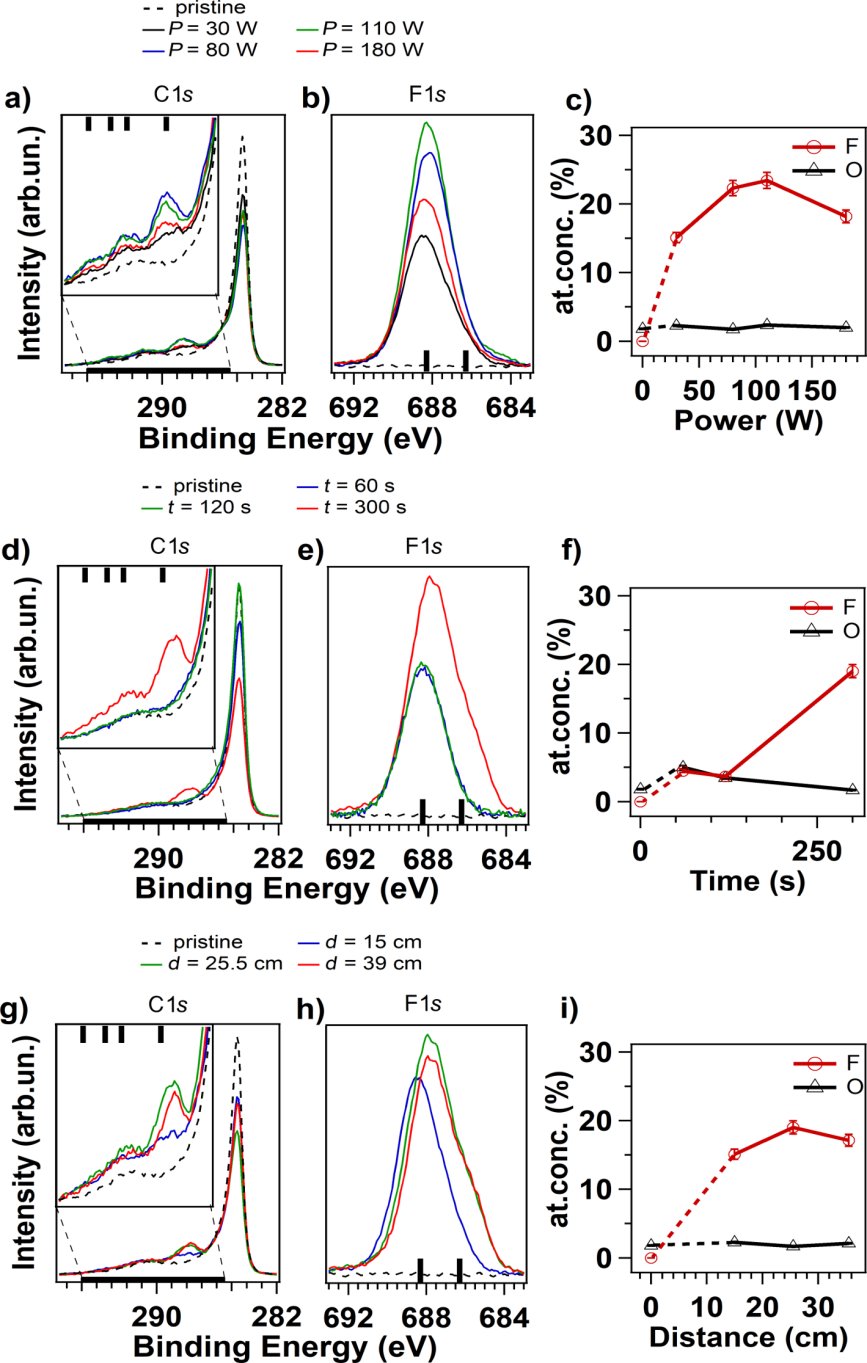
C 1s and F 1s core level spectra acquired from fluorinated vCNT after plasma treatments at different power (a,b), time (d,e) and distance of the sample from the plasma region (g,h). Evolution of F and O contents on various sample surfaces after plasma functionalization at different power (c), time (f) and distance (i).

XPS is used to evaluate the functionalization of the vCNT as a function of: plasma power values (fixing the time and the distance to: *t* = 300 s, *d* = 15 cm), exposure time (with *P* = 30 W, *d* = 39 cm) and distance of the sample surface from the plasma region (with *P* = 30 W, *t* = 300 s). The following power values 30, 80, 110 and 180 W are selected to determine the conditions under which large variations in the functionalization yield can be found; these values cover the same range used for the plasma diagnostic. In Figure 3, we report the XPS analysis performed on the samples immediately after the fluorination. The C 1s and F 1s core level spectra are illustrated for the selected set of plasma parameters: power (Figure 3a,b), time (Figure 3d,e) and distance (Figure 3g,h). The relative amount of fluorine and oxygen atoms grafted at the vCNT surface for the different experimental conditions are reported in Figure 3c,f,i. The relative concentration is calculated from the XPS spectra taking into account their respective photoionization cross-section, previously corrected by the inelastic mean free path of the electrons and by the transmission function of the spectrometer analyser. The C 1s spectra are characterized by an intense peak associated to the C–C bond in sp^2^ coordination (located at 284.5 eV). For the functionalized samples, a shoulder appears at about 286 eV accounting for an increased contribution of the C–C bond in sp^3^ coordination and for C atom first neighbour of fluorinated carbon atoms (as C–CF). In addition, new peaks emerge in the energy range going from 288 to 294 eV due to the covalent C–F bond formation, with contributions from covalent C–F, CF–CF*_n_*, CF_2_ and CF_3_ bond configurations [27,30]. The bars at the top axis of the frames indicate the binding energy of these peaks.

The asymmetric line-shape of F 1s core level signal is representative for the two carbon-fluorine bonds. They include a contribution around 686 eV, which corresponds to low density fluorination with individual F atoms covalently bonded to C in the basal plane and which is characterized by a dilated carbon-fluorine bond [20,31–32], and a second contribution around 688 eV corresponding to strong covalent C–F, with a significant distortion of the carbon lattice allowing full sp^3^ pyramidalisation of the fluorinated carbon [30,33]. The location of this latter component shows a slight dependence on the surroundings in the fluorination pattern that can be regulated with the sample distance to the glow (Figure 3h) and the functionalization time (Figure 3e). This observation indicates the presence of a different chemical environment on the surface that is characterized by dense fluorinated areas under these circumstances. The location of the C–F covalent bond in C 1s spectra is also affected, moving towards 289 eV in the curves of Figure 3a, relatively to high applied power, in comparison to the C 1s spectrum in Figure 3d corresponding to *t* = 300 s and to the C1s spectra in Figure 3g corresponding to higher distances (*d* = 25.5 and 39 cm).

The relative amount of fluorine atoms at the sample surface for increasing power is congruent with the observed changes in the plasma chemistry analysis. In the plasma discharge, the density of CF and CF_2_ species increases for increasing power until a reaction takes place and this competitive effect reduces the density of these species at high power. The fluorine content increases on the sample surface for prolonged exposure while the oxygen content is higher for shorter treatment. The oxygen concentration remains nearly constant and equal to the oxygen detected on the pristine samples for the other investigated plasma parameters. This result indicates that the oxygen-containing species are not grafted on the vCNT, therefore the surface contamination can be limited or even avoided if the sample is not immersed in the discharge. Furthermore, this is in agreement with the observation of the oxygen capacity in delivering fluorine atoms by rapidly reacting with fluorine-containing species in the discharge [8].

**Figure 4:**
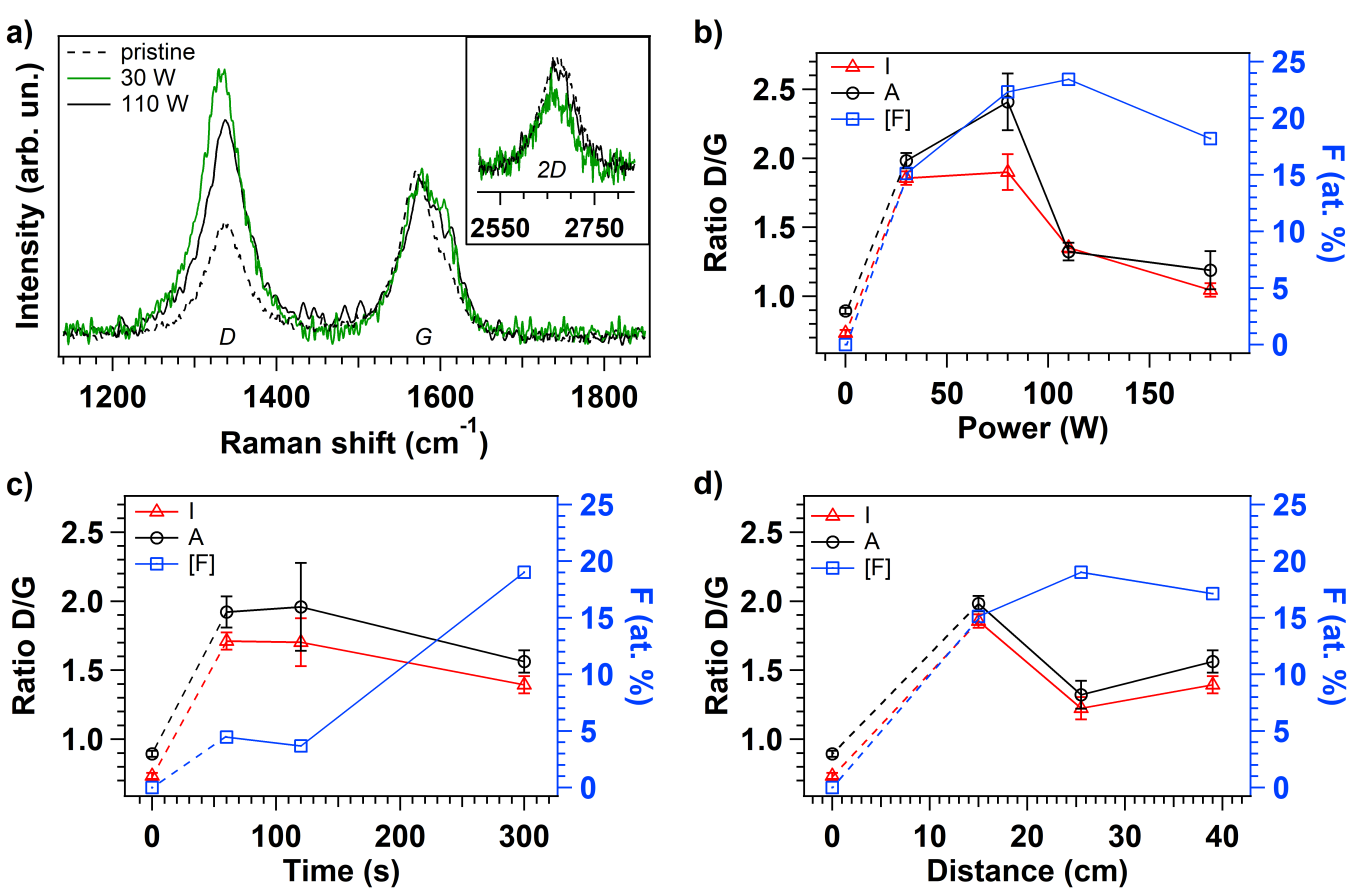
Representative Raman spectra of pristine and fluorinated vCNT (a). Evolution of intensity (*I*) and area (*A*) ratios calculated from D and G peaks in the Raman spectra for each fluorinated sample after plasma treatment at different power (b), time (c) and distance of the sample from the plasma region (d).

In order to verify the reduction in the graphitization level due to the functionalization and to the defect creation occurring at the tip of the vCNT, Raman studies are performed on the fluorinated samples. Representative Raman spectra of pristine and fluorinated vCNT, that are functionalized applying 30 and 110 W respectively, are shown in Figure 4a. The Raman modes are used as a diagnostic of disruptions in the lattice of carbon nanomaterials because the tangential mode G (located around 1570 cm^−1^) is a fingerprint of graphitic system representing the intact hexagonal crystal lattice, while the D mode (around 1330 cm^−1^) is activated after the conversion of the sp^2^ coordinated carbon lattice into sp^3^ hybridized system as a consequence of the symmetry-breaking introduced by the functionalization and by the defects [34–35]. The intensity (*I*) and the area (*A*) ratios of the D and G modes (D/G) are extracted by curve fitting procedure using Lorentzian functions to fit the peaks; a representative curve fitting result is reported in Figure S3 in Supporting Information File 1. According to the previous works [36–37], the defect quantification can be evaluated through the intensity ratios between the D and G bands, and the distance among defects can be estimated in the same way giving thus information on the crystalline domains. The evolution of the D/G ratios is illustrated in Figure 4b–d as a function of the plasma parameters together with the respective fluorine amount (right axis). The dependence on the power is illustrated in Figure 4b: at high power the tips are less damaged with respect to lower plasma power condition, in fact D/G is around 60% less intense when *P* > 80 W. This observation can be explained considering that at high power we observe a decreased production of CF_3_ fragments leading to a higher fragmentation rate (Figure 2a). As a consequence, smaller and lighter radicals and ions reach the sample surface and a limited damage is revealed compared to the functionalization under low power conditions. The evolution of the D/G ratio for increasing exposure time at a fixed power can be explained assuming that defects are also created during the plasma treatment: at the onset of the treatment the defects are not saturated with fluorine-based functionalities however they are saturated by O atoms when exposed to air, as confirmed by the higher oxygen signal detected on the surface of this sample (Figure 3f). For increasing functionalization time, the defective sites created at the vCNT surface are saturated by fluorine atoms, in agreement with the corresponding increased F content detected on the surface (blue data). The influence of the distance between the sample and the plasma region is also inferred allowing the inspection of the variable density of energetic species. Comparing the results using different distances, the D/G ratio is about 30% higher when the distance is reduced to 15 cm from the discharge while a similar relative F content is observed at the vCNT surfaces independently of the sample location. All these evidences confirm that, for short distance, a major contribution to the high intensity of the D mode comes from the defective sites rather than from the effective covalent functionalization.

**Figure 5:**
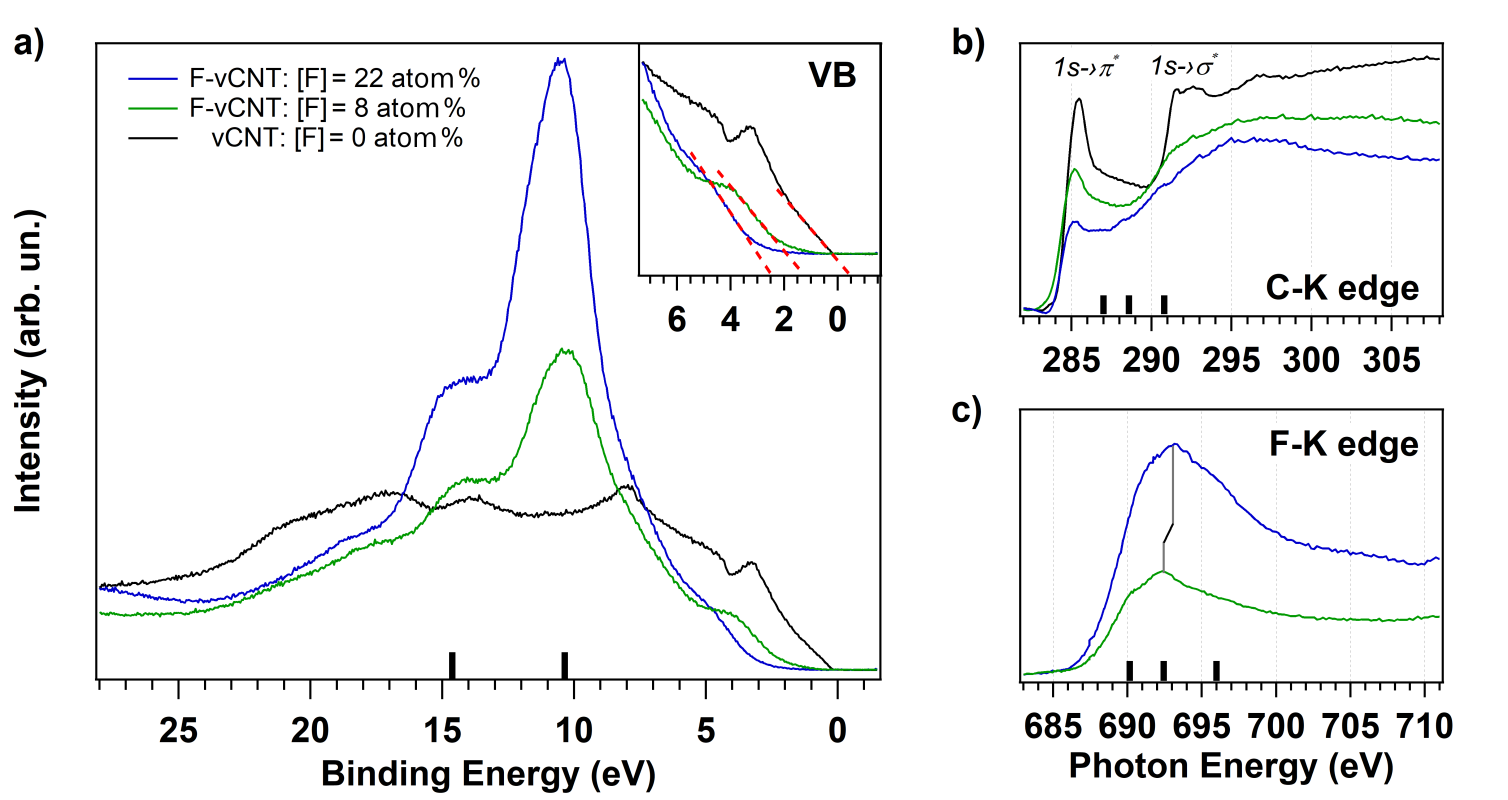
(a) Valence band spectra acquired from pristine (black line) and fluorinated vCNT with different fluorine content (22 atom %, blue line, and 8 atom %, green line). A close-up of the region near the Fermi level is depicted in the frame. For the same conditions, the absorption spectra acquired at C–K edge and F–K edge are reported in b) and c), respectively.

Furthermore, the effect of the temperature is inspected by selecting the highest fluorinated vCNT sample and heating it at *T* = 350 °C both to investigate the desorption behaviour of the high density fluorine functionalities from the carbon lattice and to consequently tailor the electronic properties [27]. The relative fluorine concentration decreased from 22 to 8 atom % after heating, the concentrations are evaluated from the F 1s and C 1s core level spectra shown in Figure S4 in Supporting Information File 1. The valence band spectra are reported in Figure 5a for the pristine (black curve) and for the fluorinated vCNT before and after heating (blue and green curves, respectively). In the binding energy range extending from 28 eV to the Fermi level (corresponding to 0 eV), the projected density of states of the pristine sample is dominated by C–C π state appearing around 3 eV, together with the C–C σ state placed around 8 eV and the σ–π hybridized states that extend from around 10 eV towards higher binding energy values. The valence band spectra of highly fluorinated sample are conversely dominated by the high cross section of the F 2p valence states, whose contribution strongly attenuates the pristine electronic structure from the Fermi level up to 20 eV. In particular, we can assign the intensity of the peak around 10 eV and at 15 eV to the F 2p-like states and to the F–C 2s bonding orbitals, respectively [19,27]. The fluorine grafting drastically reduces also the density of states just below the Fermi level. A close view of this region is illustrated in the frame, where the dotted red lines are the linear functions used to estimate the top of the valence states. These lines indicate the change of the metallic nature of the pristine sample towards the characteristic semiconducting feature of the fluorinated vCNT with a valence band offset (VBO) of more than 2 eV. The VBO is reduced to about 1.3 eV when the fluorine content at the sample surface is 8 atom %, indicating the possibility to tune the threshold energy. As a consequence of the fluorine desorption, the fluorine-related states are less dominant, in fact, the relative intensity of the density of states nearby the Fermi level increases again, due to the partial restoration of the delocalized charge in the conducting π orbitals. This effect is also observed in the near-edge absorption spectra acquired at the C–K edge (Figure 5b) where the peak at 285.4 eV (corresponding to the transition C 1s level to the unoccupied π* states, black curve) is strongly quenched because of the covalent fluorine grafting (blue curve), but its intensity is partially restored through thermal desorption (green curve). The states corresponding to the transition from C 1s to σ* are broaden due to the formation of C–F bond with σ symmetry [38], confirming the depletion of the pristine graphitization level previously observed in Raman spectra (Figure 4). Additional peaks appear at 287, 288.6 and 290.8 eV and they are more evident for high fluorine content (blue curve), as indicated by the black bars over the photon energy axis. These features are indicative for covalent bond formation [31,38] and, in correspondence to them, we can observe the presence of broad peaks in the F–K edge at 690.2, 692.4 and 696 eV photon energy. These resonances correspond to the excitations from the F1s to σ^*^ states due to the covalent interaction between F and C atoms. The black bars over the energy axis of Figure 5c are guide to locate these contributions. In particular, the peak at 692.4 eV moves towards 693.1 eV for higher fluorine coverage: this is coherent with the observed shift in the XPS spectra and it validates the presence of both different surrounding and interatomic distances when comparing the functionalized vCNT [39].

**Figure 6:**
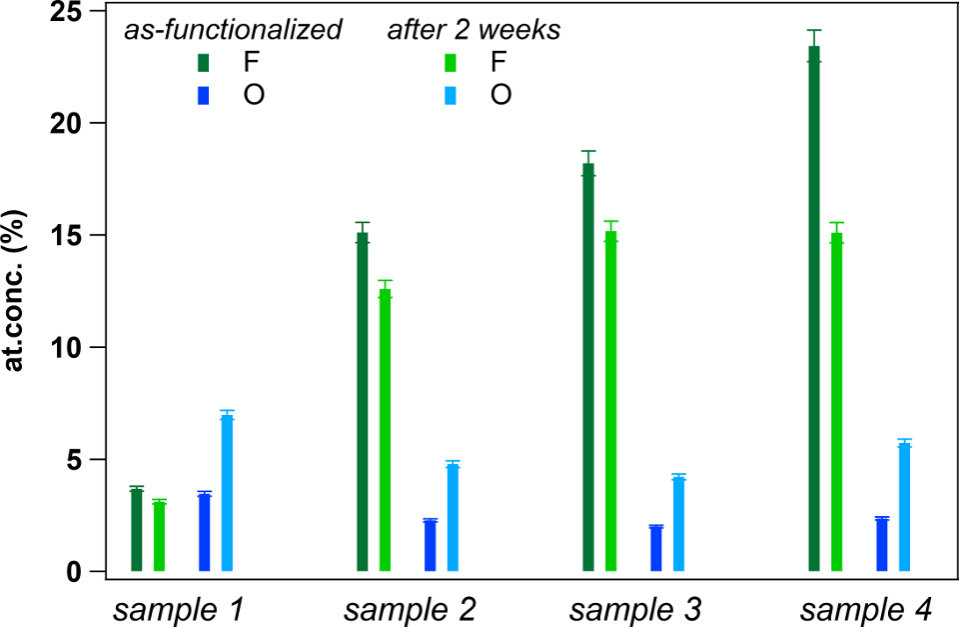
The stability of the fluorination is presented as a function of time for four different samples containing increasing amount of fluorine grafted at the surface. Green and blue colours represent the fluorine and the oxygen amounts calculated by XPS, respectively. Lighter shades are used to represent the contents after storing the samples two weeks in ambient condition.

A set of four samples, being representative of all fluorine amounts, is finally selected to verify the possibility of ageing effects, the parameters used to functionalize these samples are listed in Table S1 in Supporting Information File 1. In Figure 6, we show the evolution of the F (green) and O (blue) concentrations from the as-functionalized vCNT (dark shades) to the samples after being stored two weeks in ambient conditions (light shades). The loss of fluorine is limited to few percent in all samples except for the highly fluorinated one (sample 4). However, a tendency to saturation towards a value of about 15 atom % of fluorine atoms grafted at the surface is observed, suggesting a stable configuration for C_6.7_F when storage is performed in ambient condition. In parallel, the O uptake is limited to few percent for all samples being restricted to about 5 atom %.

## Conclusion

Plasma fluorination of vertically aligned multiwalled carbon nanotubes (vCNT) is investigated; the effect of the functionalization on the electronic properties is correlated to the CF_4_ plasma chemistry. In situ RGA mass spectrometry and Fourier transform infrared spectroscopy (FTIR) analysis are used for the plasma phase study, while the fluorinated vCNT surfaces are investigated using X-ray photoelectron (XPS) and Raman spectroscopies. For plasma power higher than *P* = 80 W, a considerable fragmentation of the parent CF_4_ molecule is observed, while the presence of contaminants in the background pressure and on the walls of the chamber are responsible for the CO, CO_2_ and COF_2_ production due to reactions occurring within the discharge. Although an ion current is measured in the mass spectrum relatively to the COF ionized molecule, FTIR analysis excludes the presence of its vibrational modes hence the presence inside the glow discharge. The combination of XPS and Raman results with the plasma chemistry analysis allows correlating the lower amount of defective sites at the CNT surface to the fragmentation of the parent molecule in lighter ions. For short treatment, non-saturated defective sites are created and a subsequent increased oxygen uptake is observed due to air exposure; on the contrary, these are available for further fluorination when the sample is exposed to the plasma for longer time, in agreement with the corresponding increased F content detected on the surface. The chemical composition of functionalized vCNT obtained by XPS confirms the absence of contaminants grafted on the sample surface when the sample is not immersed inside the discharge. The evolution of the electronic properties of fluorinated vCNT is discussed as a function of the fluorine content and ageing effects are evaluated after storing the samples for two weeks under ambient conditions: a limited loss of fluorine functionalities is observed for most of the samples.

## Experimental

Vertically aligned carbon nanotubes (vCNT) are produced by catalytic chemical vapor deposition (CCVD) at atmospheric pressure. The catalysts are prepared by magnetron sputtering: a 30 nm Al_2_O_3_ buffer layer is deposited on Si wafers with native SiO_2_ and a 6 nm Fe layer is then deposited to form nanoparticles which catalyse the vCNT growth. Then, the substrate is placed inside the reactor and heated to 750 °C at atmospheric pressure under Ar flow (120 sccm), next an additional flow of H_2_ (120 sccm) is introduced. Successively, Ar is replaced by ethylene (C_2_H_4_) flow (50 sccm) for 20 min. After the vCNT growth, the oven is again filled with Ar. The as-synthesized vCNT are typically close-packed, well-aligned CNT of 150–200 µm thick, they are multiwalled CNT having on average a dozen walls. After the plasma treatments, no changes are observed in the weight and in the structure of the vertically aligned carbon nanotubes. SEM images are recorded before and after the fluorination and they are shown in Figure S5 in Supporting Information File 1.

The CF_4_ plasma is generated using a one-turn inductive water-cooled copper coil (10 cm in diameter) located inside the vacuum chamber. The water-cooled copper coil is connected to an Advanced Energy Cesar 1310 RF (13.56 MHz) power supply via a matching network (Advanced Energy VM1000A). The plasma study and the sample functionalization are performed by applying an RF power ranging from 10 to 250 W. During the functionalization, the working pressure and the precursor flow rate are respectively regulated to 10 mTorr and 10 sccm by a throttle valve controlled by a capacitive gauge. The substrate is not biased and it is kept at room temperature. The distance between the sample and the plasma region is varied from 15 to 39 cm aiming at inspecting the difference in the resulting functionalization since the density and the chemical nature of the plasma products that reach the sample surface depend on the distance to the discharge.

The plasma composition is investigated by residual gas analysis (RGA) mass spectrometry using a quadrupole Hal IV PSM002 supplied by Hidden Analytical. The mass spectrometer is connected to the plasma chamber and it is located at around 30 cm away from the coil. Neutral species entering the mass spectrometer are ionized by electron ionization (EI) using an electron kinetic energy fixed at 20 eV to avoid any further fragmentations in the ionization source. The multiplier, the first dynode, and the current of emission are fixed at 2700 V, −1200 V, and 350 μA, respectively. The detected ions are discussed in terms of relative intensities, calculated on the basis of *I* = *I*_i_/∑_i_*I*_i_, where *I*_i_ is the raw intensity of the signal corresponding to the mass-to-charge ratio i.

Plasma products are also analysed by Fourier transform infrared spectroscopy (FTIR). The spectra are recorded using a Varian FTIR-670 spectrometer. The multipass cell consists of a cylindrical section of 120 cm length and 25 cm in diameter, crossing the chamber. One side of the section is connected to the FTIR spectrometer through a KBr window, while the other side is closed by a gold mirror. The IR beam coming from the MIR source crosses the KBr window and the plasma phase to reach the opposite side of the section and be reflected by the mirror. After reflection, the IR beam crosses again the plasma region to be collected by the detector behind the KBr window allowing an in situ multi-reflection FTIR spectroscopy with 26 passes through the plasma region corresponding to an optical length of around 31.2 m. The data reported here are obtained with a resolution of 0.2 cm^−1^.

The chemical composition of the fluorinated vCNT is calculated by XPS. The experimental geometry of the data collection allows the analysis of the tip of the vCNT, this region of the sample is denoted as the surface. The chemical composition is studied using a VERSAPROBE PHI 5000 from Physical Electronics, equipped with a monochromatic Al Kα X-ray source. The X-ray photoelectron spectra are collected at the take-off angle of 45° with respect to the electron energy analyser. The energy resolution is 0.5 eV. For the compensation of built-up charge on the sample surface during the measurements, a dual beam charge neutralization composed of an electron gun (≈1 eV) and an Ar ion gun (≤10 eV) is used.

The valence band (VB) spectra are measured with 140 eV photon energy in the ALOISA experimental chamber at the Elettra synchrotron radiation facility (Italy) [40]. The base pressure is within 10^−10^ mTorr range. Photoelectrons are collected at normal emission with the hemispherical analyser in constant pass energy mode 10 eV, the overall energy resolution is 120 meV. The carbon (C) and fluorine (F) K-edge NEXAFS spectra are obtained in the partial electron yield mode. The NEXAFS spectra are collected at the magic angle where the measured intensity distribution is independent from the molecular orientation.

The structural changes as a result of functionalization are evaluated by Raman spectroscopy. The Raman spectra are collected using a Senterra Bruker micro-Raman system spectrometer with a laser wavelength of 532 nm as excitation source. The micro-Raman system provides a spectral resolution of 3 cm^−1^. The laser power impinging on the sample is kept constant at 2 mW to avoid heating and a 50× objective is used. Five measurements are acquired at different locations on each sample and averaged. The Raman peaks are fitted using Lorentzian functions after baseline correction.

## Supporting Information

File 1 Additional experimental data.
Supporting Information File 1 features the ion signals detected in the mass spectrum corresponding to ionized contaminant molecules and shows the full range FTIR spectra. A representative curve fitting for the Raman data analysis, additional XPS spectra and SEM images are also provided.
