# Supplementary material for: Fluorination of vertically aligned carbon nanotubes: from CF4 plasma chemistry to surface functionalization
Source: Beilstein J Nanotechnol. 2017 Aug 21;8:1723–33. doi: 10.3762/bjnano.8.173 (PMC5588556; doi:10.3762/bjnano.8.173)
Supplement: File 1 — Additional experimental data. Supporting Information File 1 features the ion signals detected in the mass spectrum corresponding to ionized contaminant molecules and shows the full range FTIR spectra. A representative curve fitting for the Raman data analysis, additional XPS spectra and SEM images are also provided. [file Beilstein_J_Nanotechnol-08-1723-s001.pdf]

Supporting Information

for

Fluorination of vertically aligned carbon nanotubes: from CF_4 plasma chemistry to surface functionalization

Claudia Struzzi[1*], Mattia Scardamaglia[1], Jean-François Colomer[2], Alberto Verdini[3], Luca Floreano[3], Rony Snyders[1,4] and Carla Bittencourt[1]

Address: [1]Chimie des Interactions Plasma-Surface, CIRMAP, University of Mons, 7000 Mons, Belgium, [2]Research Group on Carbon Nanostructures (CARBONNAGe), University of Namur, 5000 Namur, Belgium, [3]CNR-IOM, Laboratorio Nazionale TASC, I-34149 Trieste, Italy and [4]Materia Nova Research Center, 7000 Mons, Belgium

Email: Claudia Struzzi - claudia.struzzi@umons.ac.be

* Corresponding author

Additional experimental data

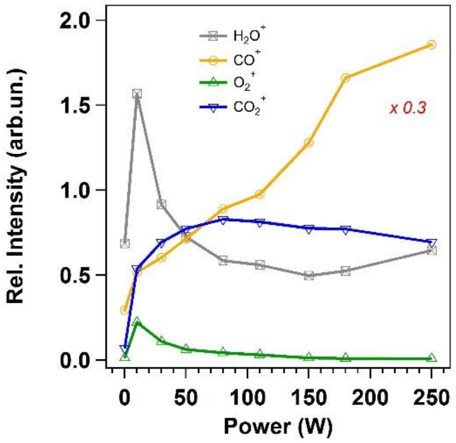

Figure S1: Evolution of the normalized ion signals extracted from the mass spectra at different plasma power (using 10 mTorr and 10 sccm). The masses under study are relative to the following ionized molecules: H_2O (*m*/*z* = 18), CO (*m*/*z* = 28), O_2 (*m*/*z* = 32), and CO_2 (*m*/*z* = 44).

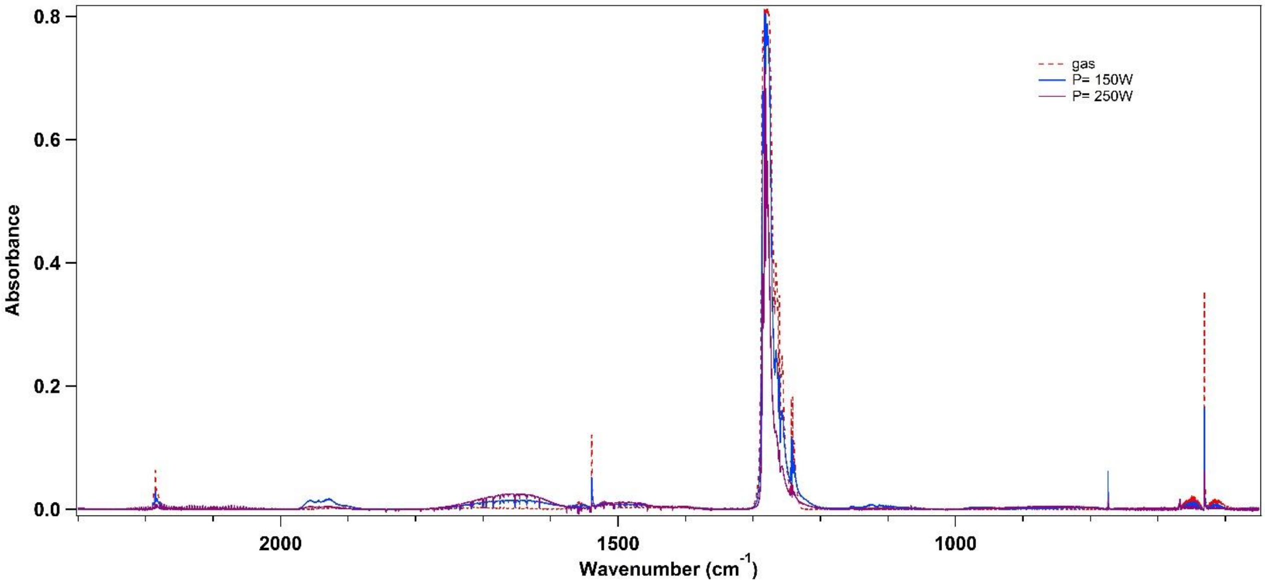

Figure S2: FTIR spectra acquired during pure CF_4 gas phase (dotted line) and during CF_4 plasma ignited at two different power values (150 and 250 W), using 10 mTorr and 10 sccm.

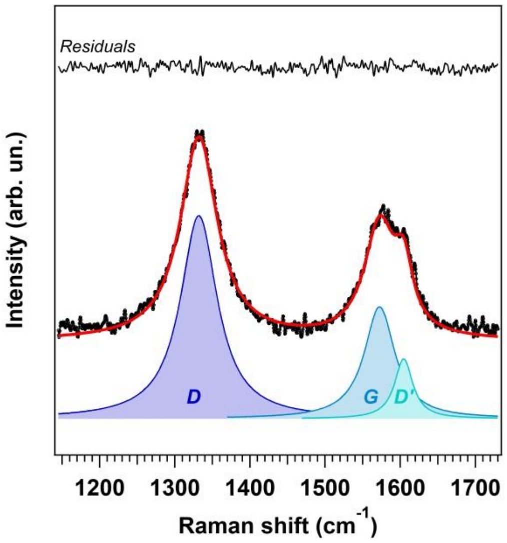

Figure S3: Representative curve fitting (red line) of Raman data (black dotted symbols) acquired from fluorinated vCNT using *P* = 30 W. Three Lorentzian components are individuated and used (D, G and D'). On top of the graph, residuals are shown.

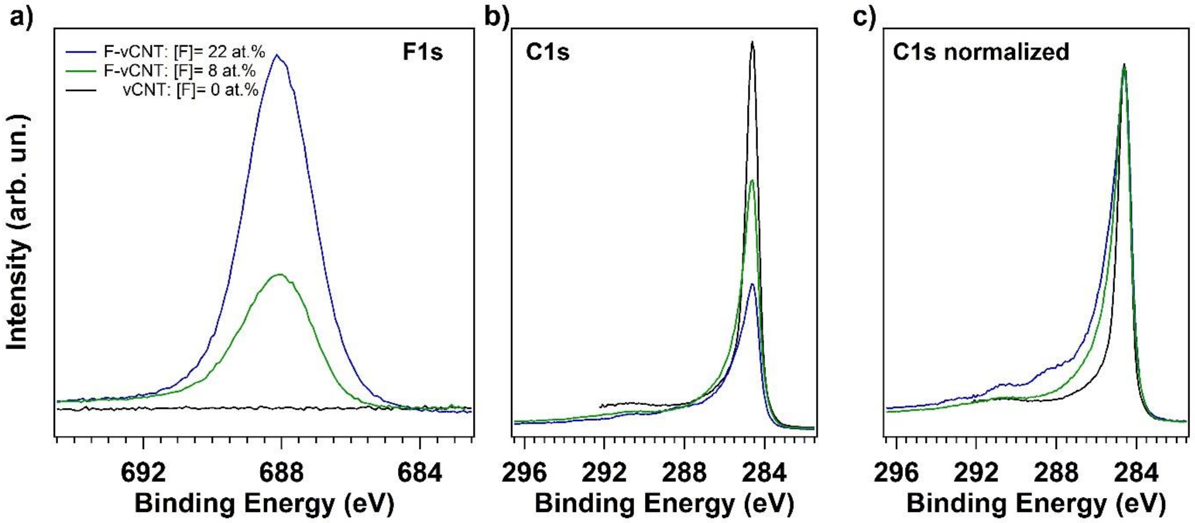

Figure S4: XPS spectra are acquired at a grazing incidence angle of 4° from the sample surface plane to enhance the surface sensitivity. The photon energy is 810 eV. a) F1*s* and b) C1*s* core level spectra acquired from pristine, and from two fluorinated vCNT containing different fluorine amount on the surface (8 atom % and 22 atom %). c) C1s normalized spectra are shown for the three samples for easy comparison of their line-shape.

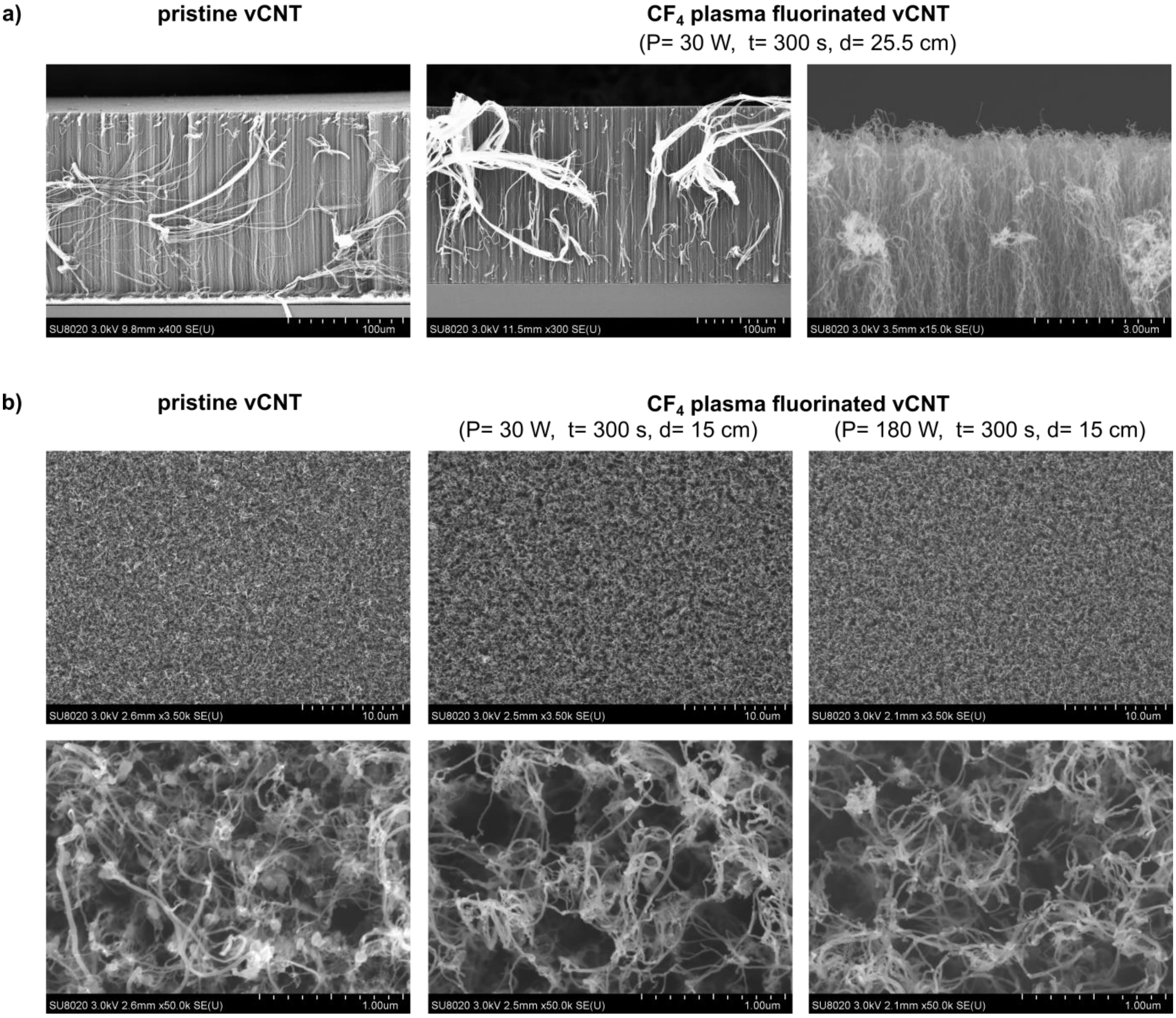

Figure S5: SEM images are acquired on pristine vCNT and on CF_4 plasma fluorinated vCNT. Side and top views are shown in (a) and in (b), respectively.

Table S1: The functionalization parameters are specified for the four samples used to test ageing effect shown in Figure 6 of the manuscript.

	P **(W)**	***t*** **(s)**	***d*** **(cm)**
sample 1	30	120	39
sample 2	30	300	15
sample 3	180	300	15
sample 4	110	300	15
